# Discovery of a Ruthenium Complex for the Theranosis of Glioma through Targeting the Mitochondrial DNA with Bioinformatic Methods

**DOI:** 10.3390/ijms20184643

**Published:** 2019-09-19

**Authors:** Le Zhang, Chen Fu, Jin Li, Zizhen Zhao, Yixue Hou, Wei Zhou, Ailing Fu

**Affiliations:** 1 College of Computer and Information Science, Southwest University, Chongqing 400715, China; zhangle06@scu.edu.cn (L.Z.); eddyblue@swu.edu.cn (J.L.); 2 College of Computer Science, Sichuan University, Chengdu 610065, China; 3 College of Pharmaceutical Sciences, Southwest University, Chongqing 400715, China; fuchen0794@swu.edu.cn (C.F.); zhaozizhen0512@hotmail.com (Z.Z.); yixue6577@163.com (Y.H.); zw2678615937@163.com (W.Z.)

**Keywords:** organometallic complexes, glioma, mtDNA mutation, computation docking

## Abstract

Glioma is the most aggressive and lethal brain tumor in humans. Mutations of mitochondrial DNA (mtDNA) are commonly found in tumor cells and are closely associated with tumorigenesis and progress. However, glioma-specific inhibitors that reflect the unique feature of tumor cells are rare. Here we uncover RC-7, a ruthenium complex with strong red fluorescence, could bind with glioma mtDNA and then inhibited the growth of human glioma cells but not that of neuronal cells, liver, or endothelial cells. RC-7 significantly reduced energy production and increased the oxidative stress in the glioma cells. Administration of RC-7 into mice not only could be observed in the glioma mass of brain by fluorescence imaging, but also obviously prevented the growth of xenograft glioma and prolonged mouse survival days. The findings suggested the theranostic application of a novel type of complex through targeting the tumor mtDNA.

## 1. Introduction

Glioma is the most aggressive and lethal brain tumor in humans, and its poor prognosis and response to treatment make combating it a challenging task [1,2]. Current standard treatment for glioma patients is surgical removal followed by radiotherapy and adjuvant chemotherapy. Due to therapeutic resistance and tumor recurrence, efforts are ongoing to identify the new molecules that are fundamental to prevent the tumor progression and provide additional methods for the treatment of glioma patients [3]. Although diverse therapeutic strategies have been attempted to slow down glioma growth at present, the lack of glioma cell specificity is one of the main bottlenecks to develop theranostic drugs for glioma [4].

A major difference between tumor (including glioma) and normal cells is the tumor cell metabolic reprogramming, where tumor mitochondria play a crucial role in the reprogramming [5,6,7]. Accumulating evidence identifies that tumor mitochondria change their original structures and functions, in which one notable feature is the mutations of mitochondrial DNA (mtDNA) [8,9,10]. Unlike nuclear DNA, mtDNA lacks damage-repair mechanisms and mutations are susceptibile to accumulate, which alters tumor mitochondrial function that shifts from major energy supplier through oxidative phosphorylation to supply part of the energy and synthesis of tumor metabolic intermediates through reversed and truncated TCA cycle [11]. Thus, specific recognition and insertion of tumor mtDNA could obstruct tumor energy and intermediate production, which would inhibit tumor growth. Several studies have examined the potential vulnerability of tumor mitochondria through targeting mtDNA mutations, and the approach may hold diagnostic and therapeutic promises [12,13].

However, glioma-specific inhibitors that bind with tumor mtDNA are rare. Here we screen ten selected compounds of substitutionally inert, octahedral ruthenium complexes and structural analogs (RCs), which several of them have been examined in vitro showing specific DNA-binding ability [14,15,16,17,18]. Compared to other small organic molecules, the RCs exhibit rich photophysical and electrochemical properties, enabling their use as fluorescent markers, DNA footprinting agents, and electrochemical probes [19,20]. Among the complexes, a unique ruthenium complex, RC-7, that is preferentially cytotoxic to glioma cells through targeting mtDNA, is filtered out. The results suggest that RC-7 could be employed as a diagnostic agent of glioma or a possible drug for the treatment. 

## 2. Results

### 2.1. Synthesis and Characteristics of the RCs

**Figure 1:**
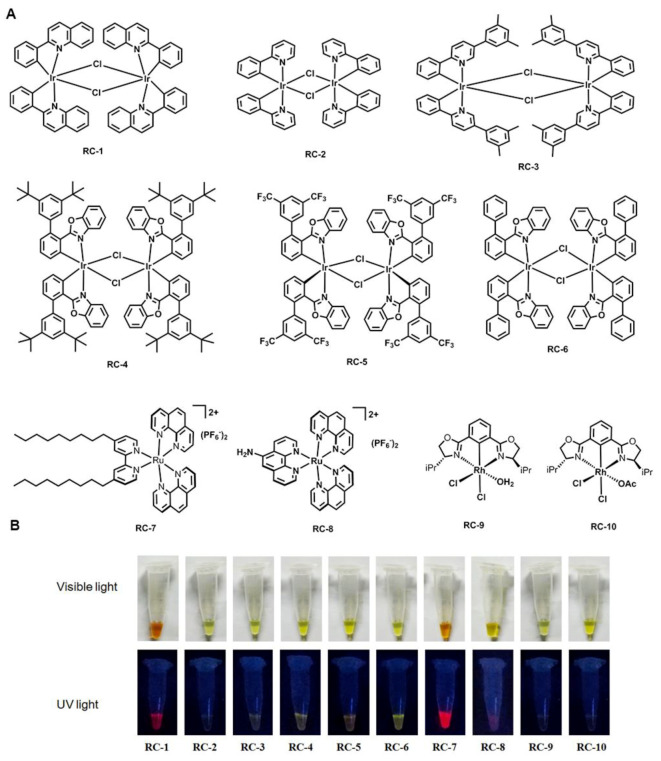
Chemical structures (**A**) and fluorescence characteristics of the RCs (RC-1~10). (**B**). The upper is the structures of RCs, and the below is the fluorescence of the RCs under visible or UV light.

The synthesis of metal complexes for bio-imaging and disease therapy has attracted considerable attention in recent years due to their specific DNA-binding ability. In this study, we screened ten biologically active and structurally representative RCs with a typical iridium chlorine-bridging dimer structure or a kinetically inert metal monomer octahedron (Figure 1A). All of the complexes showed fluorescence under ultraviolet light (UV), in which RC-1, RC-7, and RC-8 showed red fluorescence, while the others exhibited green or yellow fluorescence. The excitation and emission wavelengths (Ex/Em) of the compounds were respectively 580/620 nm (RC-1), 492/525 nm (RC-2), 492/525 nm (RC-3), 492/525 nm (RC-4), 570/590 nm (RC-5), 485/510 nm (RC-6), (550/610 nm (RC-7), 585/600 nm (RC-8), 490/510 nm (RC-9), and 490/510 nm (RC-10), evaluating by a fluorescence spectrometer. Notably, as shown in Figure 1B, RC-7 showed the strongest red fluorescence, and RC-6 exhibited the strongest yellow among the complexes. 

### 2.2. RC-7 Inhibited Cell Growth and Induced Apoptosis in U251MG Cell Line

To determine the potential capacity of the RCs that cause selective cytotoxicity, we evaluated the effect of the RCs on cell viability using different cell types (human glioma cell line U251MG, neuronal cell line CATH.a, human liver cell line L02, and human umbilical vein endothelial cells, HUVEC).

**Figure 2:**
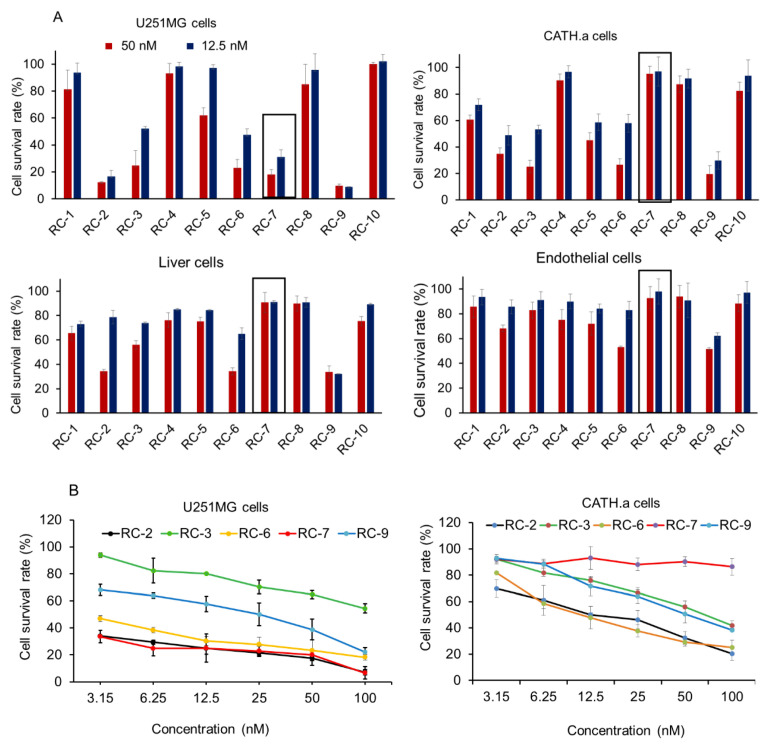
Cell viability and concentration-effect relationship of complexes RCs with different types of cells at 50 nM and 12.5 nM. (**A**) Cell viability of U251MG human glioma cells, CATH.a neuronal cells, liver cells, and human endothelial cells after RC-7 addition. The values represent the mean ± SEM of the cell survival rate. The survival rate (%) was calculated as OD (sample–blank) / (control–blank) × 100%. (**B**) Dose-response curves of U251 and neuronal cell proliferation after RC-2, RC-3, RC-6, RC-7, and RC-9 were respectively added into the cell media at the concentration of 3.15~100 nM.

As illustrated in Figure 2A, RC-7 treatment at 12.5 nM and 50 nM for 24 h resulted in a considerable reduction in cell viability in U251MG cells, higher than in the other cells tested. The result suggests that different cell types have different sensitivity to RC-7. Although U251 cells were most sensitive to RC-2 compound, RC-7 was the second most effective on U251 and had no major effect on the neuronal, liver, and endothelial cells (Figure 2).

### 2.3. The Location of RC-7 in U251MG Cells

**Figure 3:**
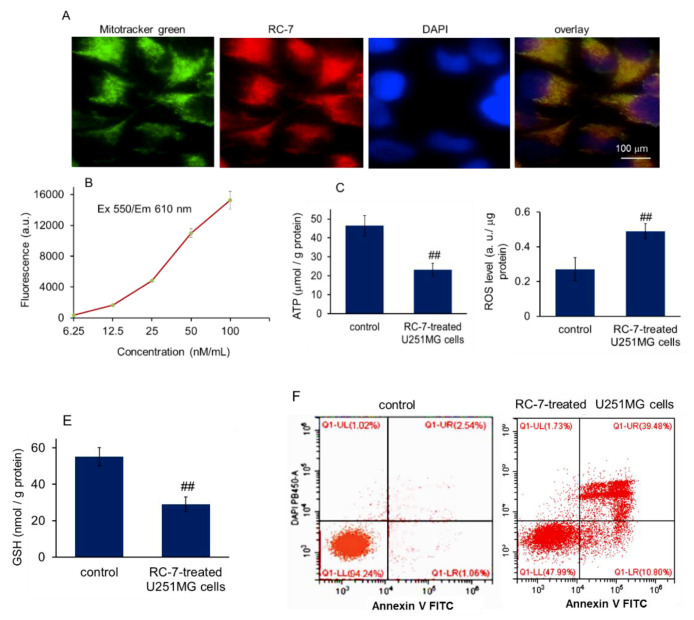
RC-7 induced U251MG cell apoptosis after arriving in mitochondria. (**A**) Intracellular distribution of RC-7 in U251MG cells. Cells were incubated with 50 nM RC-7 (red) for 2 h, followed by staining of mitochondria with MitoTracker green and the cells were observed with the confocal microscope. (**B**) Concentration-fluorescence intensity curve of RC-7 in U251MG cells. The fluorescence intensity increased in a RC-7 concentration-dependent manner. The data were expressed as mean ± SEM. Three independent experiments were performed. In addition, ATP (**C**), ROS (**D**), and GSH (**E**) were respectively determined in the RC-7 treated U251MG cells. (**F**) Cell apoptosis was measured by flow cytometry. The U251MG cells treated by RC-7 were collected and stained by Annexin V-FITC and DAPI for cell apoptosis assay.

The location of RC-7 in glioma cells was evaluated using fluorescence detection due to the auto-fluorescence of RC-7. After incubation with RC-7 for 2 h, obviously red fluorescence was observed within the U251MG cells and was mainly distributed in the mitochondria (Figure 3A), as shown by merging with MitoTracker green fluorescence (Ex/Em = 480 /520 nm). In addition, the result showed that accumulation of RC-7 in the U251MG cells was positively related to the concentration of the RC-7 at the concentrations tested (6.25 to 100 nM; Figure 3B).

Because mitochondria are mainly responsible for energy and reactive oxygen species (ROS) production, and apoptosis activation, we examined ATP, ROS, and glutathione (GSH) levels, as well as cell apoptosis after RC-7 was added into the U251MG cell media. The results showed that ATP and GSH content reduced, while ROS level increased in RC-7-treated cells (Figure 3C–E), indicating that RC-7 inhibited ATP production and increased ROS level after arriving in glioma mitochondria. In addition, approximately 50.3% of U251MG cells showed the apoptosis after RC-7 treatment by using flow cytometry detection (Figure 3F), suggesting that the mechanism by which RC-7 inhibits cell growth could be related to the induction of cell apoptosis in U251MG cells.

### 2.4. Binding of RC-7 and mtDNA

**Figure 4:**
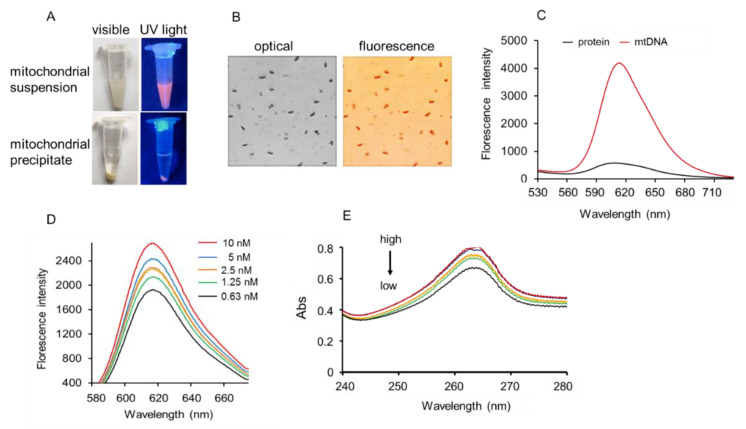
Assessment of binding of RC-7 to glioma mtDNA. (**A**) Isolated mitochondria. The suspended and precipitated mitochondria under visible or UV light. (**B**) The mitochondria were observed under confocal microscope. The mitochondria showed red fluorescence under fluorescence excitation (Ex 550 nm). (**C**) RC-7 bound mtDNA rather than mitochondrial protein extract. The RC-7-mtDNA binding fluorescence profiles were evaluated by fluorescence (**D**) and UV spectroscopy (**E**). Concentrations of RC-7 from high to low were 10, 5, 2.5, 1.25, and 0.63 nM, respectively.

Because the RC-7 could enter mitochondria of U251MG cells and specifically label the isolated mitochondria (Figure 4A,B), the binding site was further detected. It is reported that some RCs have the ability of binding with DNA [21,22], then glioma mtDNA was isolated from the U251MG cell line. After the incubation of RC-7 with the mtDNA in TE buffer (10 mM Tris, 1mM EDTA, pH 8.0), the mixture was measured by fluorescence assay, and the spectroscopic profiles of the conjugate were obtained at room temperature. The result showed that fluorescence absorption profiles (Ex = 550 nm) of the conjugate of the RC-7 and the mtDNA yielded strong fluorescence with a maximum emission centered at 610 nm (Figure 4B), suggesting that RC-7 could bind with the glioma mtDNA.

To exclude the binding of RC-7 and mitochondrial proteins, the fluorescence of the isolated mitochondrial proteins was examined after the protein extract was incubated with RC-7. Weak fluorescence intensity was observed when the mitochondrial protein extract was incubated with RC-7 (Figure 4C). The results identified that the RC-7 bound to the glioma mtDNA rather than the mitochondrial proteins. Subsequently, we investigated the binding ability of different concentrations of RC-7 to the mtDNA, and the concentration-dependent effects on the fluorescence intensity (Figure 4D) and UV absorption (Figure 4E) confirmed the specific binding of RC-7 with the giloma mtDNA.

### 2.5. Affinity Sites of RC-7 in mtDNA

**Figure 5:**
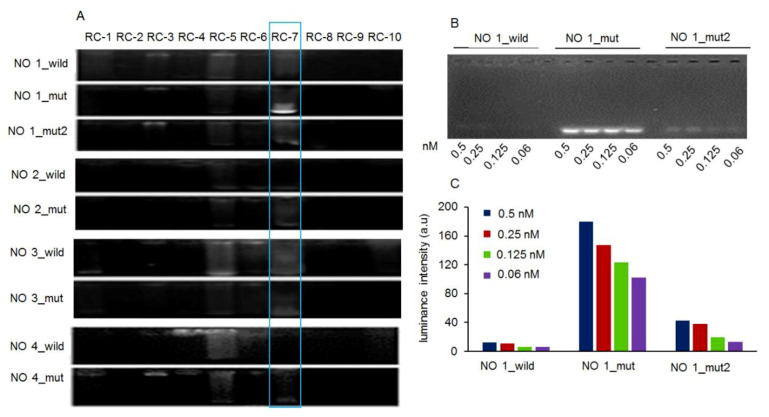
Binding of RCs and mtDNA fragments analyzed by agarose electrophoresis. (**A**) RCs were incubated with wild-type and mutant mtDNA fragments and then subjected to electrophoresis. RC-7 binding is highlighted in the rectangular box. (**B**) RC-7 (10, 5, 2.5, 1.25 nM) binding to wild-type and mutant D-loop mtDNA fragments. (**C**) The fluorescence intensity values of RC-7 binding with the dsDNA fragment containing different mutant sites were determined from three independent experiments, and the mean values were taken.

To identify the affinity sites of RC-7 in mtDNA, several mtDNA fragments and their mutant sites in glioma as reported were synthesized [23,24], in which seq. 1 comes from the regulatory region (D-loop region) of mtDNA, and seq. 2, 3, and 4 respectively are the gene fragment that encodes cytochrome c oxidase subunit I, NADH dehydrogenase subunit 3, and NADH dehydrogenase subunit 6 (Table 1). In addition, since all of the RCs exhibit auto-fluorescence, the dsDNA with bound RCs would be shown by exposure of the gel to the UV light, whereas unbound mtDNAs would not be visible in the unstained gels. Figure 5A showed that RC-7 could specifically bound to the mutant D-loop region of glioma mtDNA (seq.1_mut) but not to the wild-type region (seq.1_wild) or the region with a different mutation (seq.1_mut2; artificially designed to identify the binding specificity of RC-7 and seq.1_mut). To further determine the base selectivity of RC-7, four concentrations of RCs (10, 5, 2.5, 1.25 nM) were incubated with the wild-type and mutant dsDNA fragments. As expected, no obvious fluorescence was observed in the wild-type fragment and only a relatively weak fluorescence was observed in the seq.1_mut2 fragment, whereas a strong fluorescence appeared in the seq.1_mut at all the concentrations tested (Figure 5B). Quantification of the signals obtained from Figure 5C confirmed these results and showed that the highest binding of RC-7 to seq.1_mut occurred at the highest concentration tested (10 nM), and this binding was almost 14 and 4 times higher than that to seq.1_wild and seq.1_mut2 mtDNA, respectively, at the same concentration. The results suggest that RC-7 could recognize the seq.1_mut (57T > C) fragment of glioma mtDNA.

### 2.6. Molecular Docking of RC-7 and mtDNA Fragments

**Figure 6:**
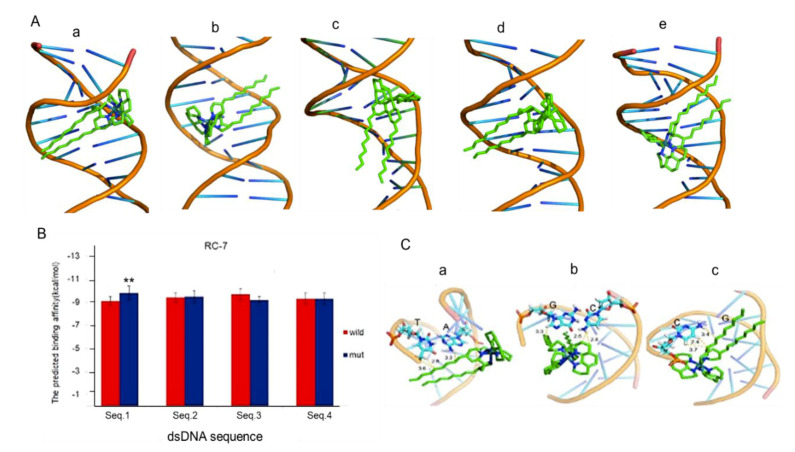
Molecular docking of RC-7 and mtDNA fragments. (**A**) Molecular docking of RC-7 with five mutant dsDNA (Table 1) by using PyMOL. (a) seq.1_mut. (b) seq.1_mut2. (c) seq.2_mut. (d) seq.3_mut. (e) seq.4_mut. (**B**) The binding affinities for RC-7 and four dsDNA sequences. Wild and mut respectively represent wild-type dsDNA and mutant dsDNA. ** *p* < 0.01 compared with the wild-type dsDNA. (**C**) RC-7 interacting with the mtDNA fragments such as (a) seq.1_wild. (b) seq.1_mut. (c) seq.1_mut2. The yellow dotted lines and values represent the three nearest distances (Å) between RC-7 and dsDNA mutation sites.

Molecular docking was performed to analyze the interaction of RC-7 and the mtDNA fragment. Figure 6A showed the molecular docking RC-7 with four mutant mtDNA fragments (seq.1_mut, seq.2_mut, seq.3_mut, seq.4_mut). Calculation of the binding affinity energy (E) of RC-7 to the different dsDNA suggested that the binding with seq.1_mut resulted in an E_mut_ statistically significantly lower than E_wild_, whereas no significant difference showed in other E_wild_ and E_mut_ binding. Moreover, Figure 6B showed that RC-7 had more tightly with seq.1_mut than to seq.1_wild.

Then the atomic interactions of RC-7 with the wild-type and mutant mtDNA in the D-loop region are calculated and compared. Figure 6C showed the insertion of RC-7 into the major groove of the mutant dsDNA. The yellow line represented the three shortest distances between atoms of RC-7 and atoms of dsDNA in seq.1_wild, seq.1_mut, and seq.1_mut2 (Figure 6C). Furthermore, the average shortest distance between RC-7 and seq.1_mut (2.9 Å) is less than those of seq.1_mut2 (3.2 Å) and seq.1_wild (3.3 Å), and the smallest binding affinity (−11 kcal/mol) and average binding affinity(−9.8 kcal/mol) of seq.1_mut is also less than those of seq.1_mut2 (−10.3 and −9.6 kcal/mol) and seq.1_wild (−9.9 and −9.1 kcal/mol) (Table 2), suggesting that RC-7 binds more tightly with seq.1_mut than seq.1_mut2 and seq.1_wild.

### 2.7. Bio-Distribution of RC-7 In Vivo

**Figure 7:**
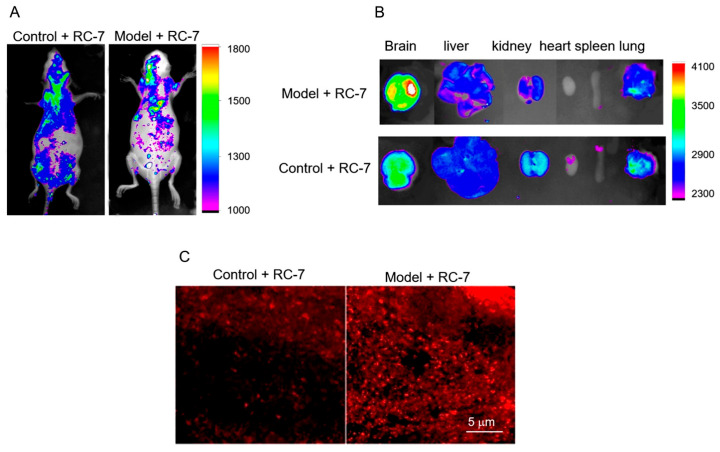
Bio-distribution of RC-7 in mice. (**A**) The distribution of RC-7 was detected with an in- vivo imaging system at 4 h after intravenous administration. (**B**) The distribution of RC-7 in important organs (the brain, liver, kidney, heart, spleen, and lungs). (**C**) Brain sections under a confocal microscope. The glioma mass showed strong red fluorescence at Ex 550 and Em 610.

Glioma-bearing mice were used to evaluate whether RC-7 could substantially accumulate in the tumor tissue after intravenous administration of RC-7. Images of the glioma-bearing mice acquired from the In-Vivo Imaging System revealed that RC-7 could enter the brain after injection, and stronger fluorescence appeared in the brains of glioma-bearing mice than those of control mice (Figure 7), which may be associated with the enhanced permeability and retention (EPR) effect of the tumor.

### 2.8. RC-7 Prolonged Survival Time of Glioma-Bearing Mice

To explore the effects of RC-7 in vivo, we examined the effects of injecting RC-7 into glioma-bearing mice. Five days after microinjection, the mice were randomly divided into three groups (*n* = 20 for each group). One group of mice used as control were microinjected with PBS into the mouse striatum (control group). The mice in the other two groups were separately intraperitoneally injected with saline (model group) and RC-7 (10 mg/kg of body weight) once a day for 7 days. At approximately 10 days after microinjection of U251MG cells, the saline-treated glioma-bearing mice exhibited glioma symptoms, including fragility, faltering, and decreased foraging behaviors, while the symptoms in RC-7-treated mice obviously improved.

**Figure 8:**
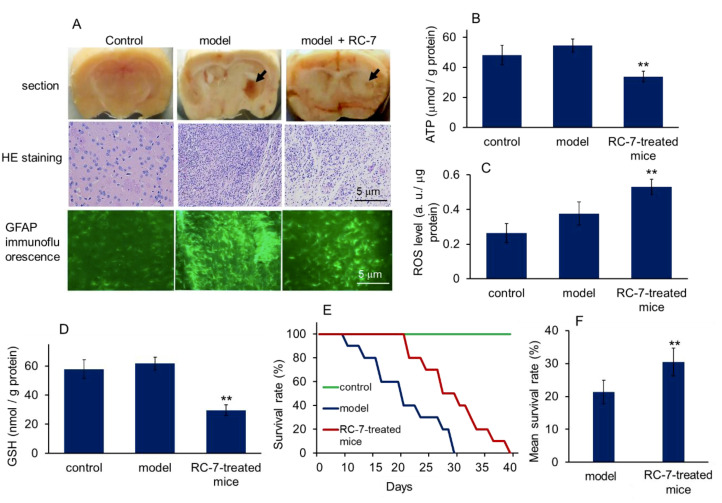
Antitumor effect of RC-7 on glioma-bearing mice. (**A**) Glioma mass in striatum. Obvious glioma mass appeared about 3 weeks after U251MG cell microinjection. The arrows indicated the glioma mass. Representative images of HE staining and GFAP immunofluorescence in mouse brain sections (400 ×) from mice microinjected with saline and treated with saline (control), mice microinjected with U251MG and treated with saline (model), and mice microinjected with U251MG cells and treated with 10 mg/kg concentration of RC-7 daily at 12 days post-microinjection and 7 days post-treatment. (**B**) ATP content, (**C**) ROS, and (**D**) GSH levels in microinjection region. (**E**) Survival curve and (**F**) mean survival days of the tumor-bearing mice untreated or treated with RC-7 (*n* = 10 for each group). **p < 0.01 compared with the model mice.

Brain sectioning and HE staining of tumor tissue were performed 12 days after microinjection. The tumor tissue displayed characteristic features of xenograft tumors, such as dense cell mass, nuclear atypia, and mitoses (Figure 8A). In addition, strong glial fibrillary acidic protein (GFAP)-positive cells appeared in the striatum of glioma-bearing mice. However, in the RC-7-treated mice, the cell mass and GFAP-positive cells were reduced, suggesting that RC-7 was effective at reducing tumor cell growth in vivo.

Moreover, we examined the ATP, ROS, and GSH content in glioma mass, and the results showed the ATP and GSH decreased, but ROS increased, in the glioma of RC-7-treated mice (Figure 8B–D), which was consistent with the in vitro study. In addition, the mice treated with RC-7 had increased survival time, compared with the untreated glioma-bearing mice (Figure 8E). Specifically, the results showed a significant increase in mean survival rate (%) in RC-7 treated mice compared to the saline-treated glioma-bearing mice, from 21 to 30 days (Figure 8F). These results indicated that RC-7 had a therapeutic effect on glioma.

## 3. Discussion

Despite many advances in the understanding and management of gliomas, these tumors are typically associated with a dismal prognosis and poor quality of life. Thus, there is a critical need to identify the differences between tumor and normal cells and to establish new diagnostic and therapeutic strategies for the disease. Although genetic and cellular studies have identified many molecules implicated in the initiation and progression of malignant gliomas, most of these molecules are related to mutations in the nuclear genome, and relatively less attention focuses on mitochondrial genome alterations in the past years. Recently, mitochondria have generated tremendous interest in the field of cancer research, since mitochondria are re-recognized as the crucial organelle for tumor cell survival and death [25,26]. Tumor mitochondria involve metabolic reprogramming to allow tumor cells to rapidly proliferate in the anoxic and acidic microenvironment, in which diverse mutations in mtDNA appear to meet the change of mitochondrial functions [27,28]. Mitochondrial functions that are dependent upon the intact of mtDNA, since mtDNA encodes 13 key subunits of the electron transfer chain. The regulatory region of mtDNA is the noncoding region that is essential for the replication and transcription of mtDNA. The accumulation of point mutations in this region predisposes cells to cancerous transformation and progress [29,30].

It is reported that complexes have the ability to target and bind mutant base pairs of nuclear DNA with high affinity and selectivity. Among these complexes, metal complexes have unique characteristics of specific base-recognizing ability and stable fluorescence. For examples, Rhodium complexes bearing the sterically expansive 5,6-chrysene diimine (chrysi) ligand could preferentially target thermodynamically destabilized mismatches [31,32],and several ruthenium complexes can be used as luminescent light switches for single-base mismatches of dsDNA [33,34]. Therefore, in this study we use the ruthenium complexes to investigate their selective toxicity to glioma cells. The experimental results turn out that RC-7 enters the cells and locates in mitochondria, and then the mitochondria are indicated stably red fluorescence even after isolated from the cells. Furthermore, we find that RC-7, bearing sterically expansive polyalkyl planar ligands, could specifically bind with mtDNA, and the mutant 57T > C in the regulatory region of mtDNA may be its affinity site, evaluated by gel electrophoresis and the molecular docking. It is assumed that RC-7 could insert into the dsDNA groove of cytosine and three repeated thymine bases of the mutant 57T > C. The results suggest that RC-7 may be used as a specific fluorescent probe to diagnose the mtDNA mutation.

After binding with the regulatory region of mtDNA, RC-7 would block the mitochondrial RNA transcription and then protein biosynthesis, which induces energy production disturbance and consequently prevents cell proliferation. Also, selectively inhibition with chemicals that bind to the base mutant site of DNA reduces the genome stability, which causes ROS increase and cell apoptosis [35,36]. For example, Bailis et al. reported that the rhodium complex [Rh(chrysi)(phen) (PPO)]^2+^ (Rh-PPO) could bind a DNA mutation to induce a lesion and thus, trigger selective cytotoxicity against mutant cells [37]. In this study, the results demonstrate that RC-7 also exhibit preferential cytotoxicity toward glioma cells, and induces oxidative stress and cell apoptosis after entering the mitochondria. The distinct cytotoxicity would make the metal complex a novel type of specific inhibitors of tumor growth.

The in vivo studies identify that RC-7 could accumulate in the glioma mass of glioma-bearing mice, evaluated by fluorescence imaging of animals and tissues, which may be associated with the passive targeting due to the EPR effect of tumors. The glioma mass shrinks after RC-7 administration and the survival days are prolonged. No obvious toxicity is observed in mice during the 7 days’ administration, implying that RC-7 might be safe for animal application.

In summary, mitochondria are not only a powerhouse responsible for cell survival but also a suicidal weapon that regulates cell death. Specific recognition and insertion of tumor mtDNA could prevent the energy and substance metabolism of the tumor and then halt tumor proliferation but not affect other cells. This study uncovers a ruthenium complex RC-7, which has the selective base-recognizing ability in glioma mtDNA, and then leads to glioma cell apoptosis and prolongs the survival days in the glioma-bearing mice. The study, at the first time, finds a novel type of chemical for glioma theranostics and provides an anti-glioma approach based on glioma mtDNA.

## 4. Materials and Methods

### 4.1. Synthesis of RCs

**Figure 9:**
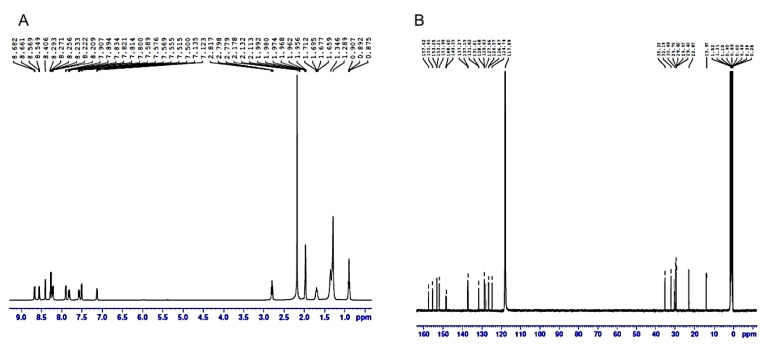
The NMR spectra of RC-7. (**A**). ^1^H-NMR spectrum. (**B**), ^13^C-NMR spectrum.

All reactions were carried out under nitrogen atmosphere. All reagents were purchased from Alfa, Aldrich, and used without further purification. Column chromatography was performed with silica gel (230~400 mesh). ^1^H- and ^13^C NMR spectra were recorded on a Bruker AVANCE (400 MHz) spectrometer at ambient temperature. NMR standards used were as follows: (^1^H NMR) CD_3_CN = 1.94 ppm; (^13^C NMR) CD_3_CN = 1.32 ppm. High-resolution mass spectra were recorded on a Finnigan LTQ-FT instrument using the ESI technique. The structures of the RCs were shown in Figure 1. RC-1–6 and 8–10 were synthesized according to the published procedures [38,39,40]. The synthetic procedure for RC-7 is as follows. In a closed brown vial fitted with a septum, a solution of cis-[Ru(phen)_2_Cl_2_] (1.0 eq), C_9_-bpy (2.0 eq) in ethylene glycol/water (V:V = 1:1) (20 mM) was degassed with argon and then heated at 140 °C (oil bath temperature) for 1 h. To remove ethylene glycol and facilitate purification, the reaction mixture was precipitated by the addition of excess solid NH_4_PF_6_ after cooling to room temperature. Then the crude product was filtered and collected, then dissolved in CH_3_CN and subjected to silica gel chromatography (eluent: CH_3_CN, CH_3_CN:H_2_O:KNO_3_ (sat) = 100:3:1, CH_3_CN:H_2_O:KNO_3_ (sat) = 50:3:1). The product eluents were concentrated by rotary evaporator and dissolved in minimal amounts of water or ethanol/water (V:V = 1:1). Subsequently, the solution was precipitated by the addition of excess solid NH_4_PF_6_. The precipitate was filtered and washed twice with water, and then dried under high vacuum to afford the product with yield of 80%. ^1^H-NMR (400 MHz, CD_3_CN): *δ* (ppm) 8.68 (d, *J* = 6.0 Hz, 1H), 8.55 (d, *J* = 6.0Hz, 1H), 8.41 (s,1H), 8.21~8.29 (m, 3H), 7.90 (d, *J* = 5.7 Hz, 1H), 7.82 (dd, *J* = 6.1, 5.7 Hz, 1H), 7.57 (dd, *J* = 6.0, 5.5 Hz, 1H), 7.51 (d, *J* = 5.9 Hz, 1H), 7.13 (d, *J* = 6.2 Hz, 1H), 2.80 (dd, *J* = 5.6, 2.1 Hz, 2H), 1.68 (dd, *J* = 6.1, 2.0 Hz, 2H), 1.29~1.35 (m, 12H), 0.89 (t, *J* = 5.7 Hz, 3H) (Figure 9A). ^13^C-NMR (100 MHz, CD_3_CN): *δ* (ppm) 157.62, 155.45, 153.25, 153.21, 151.98, 148.60, 148.35, 137.27, 137.15, 131.62, 131.61, 128.68, 128.63, 127.96, 126.57, 126.47, 124.74, 35.33, 32.19, 30.49, 29.75, 29.60, 29.57, 29.40, 22.97, 13.97 (Figure 9B). HRMS calcd. for C_52_H_60_F_6_N_6_PRu (M-PF_6_) + : 1015.1282, found: 1015.1298. Moreover, the excitation and emission wavelengths (Ex/Em) of the compounds were respectively detected by using a fluorescence spectrometer (Thermo-Fisher Sci. Ltd. Co., USA).

### 4.2. Cell Culture

U251MG human glioma cells, CATH.a neuronal cells, L02 human liver cells, and human umbilical vein endothelial cells (HUVEC) were stored in the laboratory and originated from ATCC (Manassas, Virginia, USA). The cells were grown in Dulbecco’s modified Eagle’s medium (DMEM) supplemented with 10% fetal bovine serum (FBS), 100 units/mL penicillin, and 100 μg/mL streptomycin and incubated at 37 °C and 5% CO_2_ in a humidified incubator (CCL-170B-8, ESCO, Timur, Indonesia). All cell culture reagents were obtained from GIBCO (Life Technologies, Carlsbad, California, USA).

### 4.3. Cytotoxicity Assay

The cytotoxicity of the RCs to the cells was evaluated by assessing their metabolic activity using the MTT assay. Briefly, the cells were seeded in a 96-well plate for 24 h and then treated with RCs for another 24 h. The cells were washed with PBS (pH 7.4), 20 μL of MTT (5 mg/mL; Beijing Beyotime Biotech. Co., Beijing, China) was added to each well, and the cells were incubated for another 4 h. After the medium was discarded, 100 μL of DMSO was added, and the absorbance was measured at a 570 nm wavelength on a microplate reader (Model 680, Bio-Rad Laboratories Inc, Hercules, CA, USA). The cell viability was represented as OD (sample – blank) / OD (control –blank) × 100%.

The cell apoptosis also was examined. The cells were seeded into a 12-well plate for 24 h and treated with RCs for 24 h. Then the cells were digested with 0.25% trypsin and washed by PBS for 3 times. Cell apoptosis was evaluated using Annexin V-FITC in a flow cytometer (BD Biosciences; USA).

### 4.4. Cell Uptake and Biochemical Assay

The U251MG cells were cultured in 24-well plates for 24 h at 37 °C. When the confluence of the cells reached 60%, the media were replaced with fresh medium. RCs were added to the medium at a final concentration of 50 μM. After incubation for 30 min, the cells were washed three times with fresh medium, MitoTracker (a mitochondrial indicator) was added to the medium, and the cells were incubated for another 30 min. The cells were observed under a confocal microscope (ZEISS, Oberkochen, Germany) with the green, red, and blue channels according to the manufacturer’s protocol. Additionally, the fluorescence intensity of the RCs in cells was measured by using a fluorescence microplate reader (TECAN infinite M200 PRO, Männedorf, Switzerland). Moreover, the levels of ATP, GSH, and ROS were respectively determined by using commercial kits (Nanjing Jiancheng Biotech. Ltd. Co., Nanjing, China), in which ATP level was measured by spectrophotometer method, and DCFH-DA (Sigma-Aldrich Co. Missouri, USA) and 5,5-dithiobis(2-nitrobenzoic acid) (Sigma-Aldrich Co. Missouri, USA) was respectively used to determine ROS and GSH content. Three independent experiments were performed for each assay.

### 4.5. Isolation of Mitochondria, mtDNA, and Protein Gradients

U251MG cells were digested with 0.25% trypsin and then washed three times with PBS (pH 7.4). The cells were collected, and then mtDNA and mitochondrial protein preparation were carried out at 0~ 4 °C [41]. Briefly, cells in SE buffer (0.25 M sucrose, 30 mM Tris-HCl, 10 mM EDTA-Na_2_, 2.5 mM CaCl_2_, pH 8.0) were gently homogenized using a glass homogenizer and then centrifuged at 1500 rpm for 10 min. The supernatant was centrifuged at 12000 rpm for 20 min. The supernatant thus obtained was discarded, and the precipitate in SE buffer was further centrifuged at 12000 rpm for 20 min to obtain mitochondria. The mitochondria were observed under the confocal microscope. Then the mitochondria were suspended in SE buffer, incubated at room temperature for 15 min and then centrifuged at 12,000 rpm for 8 min. The precipitate was then suspended in buffer solution I (10 mM Tris-HCl, 1.0 mM EDTA-Na_2_, 15 M NaCl, pH 8.0), followed by buffer solution II (1% SDS, 0.2 M NaOH) and the suspension was incubated in an ice bath for 10 min to lyse the mitochondria. Subsequently, buffer solution III (2.9 mM KAc, 4% formylic acid, pH 5.5) was added to the mixture, which was incubated in an ice bath for 40 min. The supernatant was centrifuged at 12,000 rpm for 6 min and mixed with 2 volumes of anhydrous ethanol to acquire crude mtDNA, while the precipitate was collected as the protein extractfor use. The crude mtDNA was dissolved in TE buffer (50 mM Tris-HCl, 10 mM EDTA-Na_2_,pH8.0), and RNase (final concentration 200 μg/mL) was added to digest the RNA at 37 °C for 30 min. Then, the same volume of water-saturated phenol was added to the solution and gently shaken for 15 min. The solution was centrifuged at 12,000 rpm for 8 min at 0 °C. Then, the precipitate (mtDNA) was dissolved in TE buffer for further use. The DNA was quantified by using NanoDrop-2000 (Thermo-Fisher Sci. Ltd. Co., MA, USA), and BCA assay was used for protein concentration measurement.

### 4.6. Binding Test of mtDNA and RC-7

Spectroscopic profiles were used to determine the binding capability of mtDNA and the RC-7. The TE buffer solution of mtDNA and mitochondrial protein extract (10 nM) were respectively combined with 10 nM RC-7 and incubated for 2 min at room temperature. The fluorescence profiles were measured at Ex of 550 nm and Em of 610 nm (same Ex/Em as the RC-7) using the fluorescence spectrometer. Different concentrations of RC-7 (10, 5, 2.5, 1.25 nM) were incubated with mtDNA, and the fluorescence and ultraviolet (UV) absorption profiles were determined using a fluorescence spectrometer and UV-viable spectrophotometer (Thermo-Fisher Sci. Ltd. Co., MA, USA).

### 4.7. Binding of RCs and mtDNA Fragments

The wild-type and mutant mtDNA fragments were synthesized by Beijing Huada Gene Ltd. Co. (Beijing, China). The fragments were dissolved in nuclease-free water to a concentration of 10 nM. Then, RC-1 to 10 (10 nM) were individually added to the dsDNA solutions for 2 min incubation at room temperature. The conjugated dsDNA/ruthenium complexes were subjected to 1.0% agarose electrophoresis in TAE buffer (0.04 M Tris-acetate, 0.001 M EDTA, pH 8.0) at 80 mV for 15 min. Gel images were taken by using Gel Doc XR (Bio-Rad, CA, USA). Moreover, the D-loop sequences were incubated with different concentrations of these complexes (10, 5, 2.5, 1.25 nM), and the intensity of the fluorescence was measured using the Gel Doc XR software.

### 4.8. Molecular Simulation of the Affinity of the Ruthenium Complexes and mtDNA Fragments

Molecular docking was used in the study to identify the RC-mtDNA binding. ChemOffice software was used to optimize the 3D structure of complexes with respect to the energy minimization [42]. Then, the binding affinities of the complexes and representative mtDNA fragments were calculated by using molecular docking software (Autodock Vina; http://vina.scripps.edu/) [43].

### 4.9. Preparation of Murine Intracranial Glioma Model

Male BALB/c nude mice aged 4–5 weeks and weighed around 20 g (SCXK [Jing 2009–0015]) were purchased from Chongqing Medical University, Chongqing, China. Animals were maintained under standard housing conditions with ad libitum access to standard laboratory mouse chow and water. Animals were used in the study according to the guidelines of the Institutional Animal Committee of Southwest University, Chongqing, and the animal experiments were approved by the Institutional Animal Committee (#2018–012).

The mice were anesthetized with an intraperitoneal injection of 30 mg/kg pentobarbital sodium and then placed on a small animal stereotaxic apparatus. U251MG cells were collected after digestion by 0.25% trypsin and washed by PBS for 3 times. Then the cells (1 × 10^7^ cells/5 μL) suspended in PBS were microinjected into the right striata of the mice at sites with the following stereotaxic coordinates: +0.26 mm relative to the bregma, 1.87 mm relative to the midline, and 3.50 mm below the skull surface [44]. The cells were injected slowly, and the syringe remained for 10 min before the needle was withdrawn. Then, the burr hole was sealed with bone wax. The mice were carefully placed into their home cages, and their condition and behavior were observed.

### 4.10. Bio-Distribution of Ruthenium Complex In Vivo

The ruthenium complex was respectively intravenously injected at the dose of 20 mg/kg body weight, respectively into the glioma model mice. Then the mice were anesthetized by isoflurane following 1 h injection. After the mice were placed on the platform of BLT In-Vivo Imaging System (BLT Photon Tech., Guangzhou, China), fluorescence images were captured according to the manufacturer’s instructions. Subsequently, the mice were euthanized by overdose pentobarbital sodium, and the brain, heart, lungs, kidneys, spleen, and liver were dissected out for fluorescence imaging. The fluorescence images were captured at an excitation wavelength of 550 nm and an emission wavelength of 610 nm.

### 4.11. Effect of Ruthenium Complex Treatment on the Orthotopic Model of Glioma

Five days after microinjection of the cells, the mice were randomly divided into two groups (*n* = 13 mice per group). One group was intraperitoneally injected with saline (model group) whereas the other was intraperitoneally injected with RC-7 (10 mg/kg body weight; model + RC-7 group) once a day for 7 days. The control group microinjected PBS into right striatum was intraperitoneally injected with saline. Then 3 mice per group were euthanized with an overdose of sodium pentobarbital, and the brains were dissected out and fixed in 4% paraformaldehyde at 4 °C, then dehydrated in a series of 10%, 20%, and 30% sucrose. The brain was continuously sectioned, and the sections were stained with hematoxylin and eosin (HE). The immunofluorescence of glial fibrillary acidic protein (GFAP), a specific marker of glial cells, was used to further identify glioma growth. Meanwhile, the remaining 10 mice per group were used to measure the survival time to evaluate the effect of RC-7 treatment on the glioma-bearing mice.

### 4.12. Statistical Analysis

All data are expressed as the mean ± SEM. The *t-*test was used to compare two groups, and ANOVA was used for multiple-group analysis. A value of *p* < 0.05 was considered statistically significant.

## Figures and Tables

**Table 1:** Mitochondrial DNA sequences that were used in the study.

Number of Sequence (gene)	mtDNA	Site	Sequence	Wild-Type/Mutant
1 (regulatory region)	seq.1_wild	57T	CTCCATGCATTTGGTATTTT	wild-type
	seq.1_mut	57T > C	CTCCATGCATTTGGTACTTT	mutant
	seq.1_mut2	57T > G	CTCCATGCATTTGGTAGTTT	mutant
2 (cytochrome c oxidase subunit I)	seq.2_wild	6340C	TAGACCTAACCATCTTCTCC	wild-type
	seq.2_mut	6340C > T	TAGACCTAATCATCTTCTCC	mutant
3 (NADH dehydrogenase subunit 3)	seq.3_wild	10261A	TTTGATCTAGAAATTGCCCT	wild-type
	seq.3_mut	10261A > G	TTTGATCTAGGAATTGCCCT	mutant
4 (NADH dehydrogenase subunit 6)	seq.4_wild	14181A	ATTACAATATATACACCAAC	wild-type
	seq.4_mut	14181A > C	ATTACAATATCTACACCAAC	mutant

Here, seq.1, seq.2, seq.3, and seq.4 represent the four double-stranded glioma mtDNA fragments. seq.1_wild, seq.2_wild, seq.3_wild, and seq.4_wild are four wild-type dsDNA fragments, and seq.1_mut, seq.2_mut, seq.3_mut, and seq.4_mut are mutant dsDNA. Additionally, seq.1_mut2 denotes mutant dsDNA (II). The shadow showed the mutant site in the dsDNA.

**Table 2:** Binding affinities (kcal/mol) and three nearest distances (Å) of RC-7 and mtDNA (57T; 57T > C; 57T > G).

mtDNA	Site	Smallest Binding Affinity	Average Binding Affinity	Three Nearest Distances between RC-7 and dsDNA			Average Distance
seq.1_wild	57T	−9.9	−9.1	2.8	3.3	3.6	3.2
seq.1_mut	57T > C	−11	−9.8	2.6	2.8	3.3	2.9
seq.1_mut2	57T > G	−10.3	−9.6	2.4	3.4	3.7	3.3
