# Correction: Zhang et al. Discovery of a Ruthenium Complex for the Theranosis of Glioma through Targeting the Mitochondrial DNA with Bioinformatic Methods. *Int. J. Mol. Sci.* 2019, *20*, 4643

**DOI:** 10.3390/ijms26136425

**Published:** 2025-07-03

**Authors:** Le Zhang, Chen Fu, Jin Li, Zizhen Zhao, Yixue Hou, Wei Zhou, Ailing Fu

**Affiliations:** 1 College of Computer and Information Science, Southwest University, Chongqing 400715, China; zhangle06@scu.edu.cn (L.Z.); eddyblue@swu.edu.cn (J.L.); 2 College of Computer Science, Sichuan University, Chengdu 610065, China; 3 College of Pharmaceutical Sciences, Southwest University, Chongqing 400715, China; fuchen0794@swu.edu.cn (C.F.); zhaozizhen0512@hotmail.com (Z.Z.); yixue6577@163.com (Y.H.); zw2678615937@163.com (W.Z.)

In the original publication [1], there was a mistake in Figure 7B as published. An incorrect image was inadvertently used (Model + RC-7 samples—7B) during the uploading of images that were of high similarity. The corrected Figure 7B appears below. The authors state that the scientific conclusions are unaffected. This correction was approved by the Academic Editor. The original publication has also been updated.

## Figures and Tables

**Figure 7:**
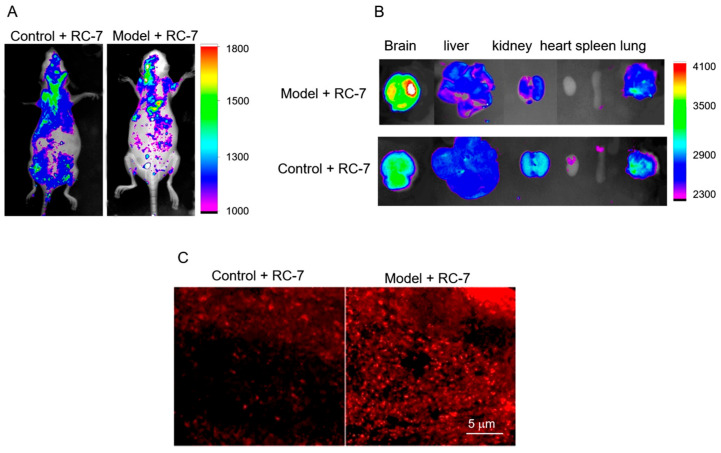
Bio-distribution of RC-7 in mice. (**A**) The distribution of RC-7 was detected with an in- vivo imaging system at 4 h after intravenous administration. (**B**) The distribution of RC-7 in important organs (the brain, liver, kidney, heart, spleen, and lungs). (**C**) Brain sections under a confocal microscope. The glioma mass showed strong red fluorescence at Ex 550 and Em 610.
